# Dynamically encircling an exceptional point in anti-parity-time symmetric systems: asymmetric mode switching for symmetry-broken modes

**DOI:** 10.1038/s41377-019-0200-8

**Published:** 2019-10-02

**Authors:** Xu-Lin Zhang, Tianshu Jiang, C. T. Chan

**Affiliations:** 1 State Key Laboratory of Integrated Optoelectronics, College of Electronic Science and Engineering, Jilin University, Changchun, China; 2 Department of Physics, The Hong Kong University of Science and Technology, Clear Water Bay, Hong Kong, China

**Keywords:** Microwave photonics, Optoelectronic devices and components

## Abstract

Dynamically encircling an exceptional point (EP) in parity-time (*PT*) symmetric waveguide systems exhibits interesting chiral dynamics that can be applied to asymmetric mode switching for symmetric and anti-symmetric modes. The counterpart symmetry-broken modes (i.e., each eigenmode is localized in one waveguide only), which are more useful for applications such as on-chip optical signal processing, exhibit only non-chiral dynamics and therefore cannot be used for asymmetric mode switching. Here, we solve this problem by resorting to anti-parity-time (anti-*PT*) symmetric systems and utilizing their unique topological structure, which is very different from that of *PT*-symmetric systems. We find that the dynamical encircling of an EP in anti-*PT*-symmetric systems with the starting point in the *PT*-broken phase results in chiral dynamics. As a result, symmetry-broken modes can be used for asymmetric mode switching, which is a phenomenon and application unique to anti-*PT*-symmetric systems. We perform experiments to demonstrate the new wave-manipulation scheme, which may pave the way towards designing on-chip optical systems with novel functionalities.

## Non-Hermitian optics: Encircling exceptional points leads to advanced wave-manipulation technologies

By steering microwaves in an anti-parity-time symmetric system, Chinese scientists have explored the topological physics and their wave-manipulation applications in optics and photonics. Recent developments in non-Hermitian systems, i.e., open systems that can exchange energy with surroundings, have given rise to new optical devices such as isolators, sensors, and absorbers. Such systems exhibit the so-called exceptional points, where two or more resonances have equal frequencies and dissipations. By sending microwaves along a waveguide with specially designed boundaries, which is equivalent to looping the waveguide resonance around an exceptional point in a parameter space, Xu-Lin Zhang from Jilin University and Che Ting Chan from the Hong Kong University of Science and Technology have demonstrated a new method for manipulating electromagnetic waves in waveguides. Their work could pave the way for on-chip optical devices with new functionalities.

## Introduction

Non-Hermitian systems obeying parity-time (*PT*) symmetry, i.e., [*PT,H*] = 0, with *H* being the non-Hermitian Hamiltonian, have attracted considerable attention in recent years^1–4^. Most of the interesting properties of non-Hermitian systems are found at the exceptional point (EP)^5,6^, which has led to many novel phenomena and plausible applications^7–17^. The topological structure of the energy Riemann surface around the EP is of great interest, and it is known that adiabatically encircling an EP can result in an intriguing “flipping of the eigenstate” phenomenon, in which an eigenstate does not come back to itself after going around a loop in parameter space^18^. This phenomenon was demonstrated experimentally^19–21^ by measuring the spectra and eigenfields at different points of a loop enclosing the EP. In contrast to these “static” measurements, in which the results at different locations are independently measured, dynamically encircling an EP where the phase information at different points is closely related is predicted to exhibit an intriguing chiral behaviour^22–27^ because of the non-Hermiticity induced non-adiabatic transitions (NATs)^28^. The chiral behaviour was recently observed experimentally in microwave waveguides^29^ and silicon photonic waveguides^30^. In the experiment, the starting/end point of the loop lies in the *PT*-symmetric phase, where the eigenmodes are symmetric and anti-symmetric modes^30^. The chiral behaviour is of great importance since it can be used for asymmetric mode switching, i.e., a robust direction-selective energy transfer scheme that has practical applications for on-chip optical devices such as optical isolators^31^. In fact, the eigenmodes in non-Hermitian systems can be divided into two classes: symmetry-unbroken modes (i.e., symmetric and anti-symmetric modes) and symmetry-broken modes (i.e., each eigenmode is localized in one oscillator only). For on-chip waveguide-based optical devices, symmetry-broken modes are of more interest since they are typically used as the input and output of the system^32,33^. However, it is demonstrated that when the starting point of the loop lies in the *PT*-broken phase where the eigenmodes are symmetry-broken modes, dynamically encircling the EP results in a non-chiral transmission behaviour^34^. Therefore, the symmetry-broken modes cannot be used for asymmetric mode switching in *PT*-symmetric systems.

Anti-*PT*-symmetric systems, the Hamiltonians of which obey {*PT,H*} = 0, have also attracted much attention recently^35–40^. Mathematically, the anti-*PT*-symmetric Hamiltonian can be obtained by multiplying the *PT*-symmetric Hamiltonian by a constant “*i*”, but it is challenging to construct a realistic anti-*PT*-symmetric system, as it requires the coupling between the two bare states to be a purely imaginary value. As such, there are very limited experimental works on anti-*PT*-symmetric systems^37,40^. Anti-*PT*-symmetric systems also possess EPs, but the different Hamiltonian may lead to different physics. Therefore, it is important to experimentally explore the unique characteristics of anti-*PT*-symmetric systems and employ the physics for novel applications, especially those that cannot be realized in conventional *PT*-symmetric systems.

In this work, we report the first experiment on the dynamical encircling of an EP in an anti-*PT*-symmetric system, which consists of three waveguides, with an absorber attached to the middle one. The two gap distances are designed to vary continuously along the waveguide direction so that the transmission of electromagnetic waves through the system is equivalent to a loop enclosing an EP in the parameter space. We discover a chiral transmission behaviour when the starting/end point of the loop lies in the *PT*-broken phase, where the eigenmodes are symmetry-broken modes. This is in contrast to *PT*-symmetric systems, where the chiral behaviour applies only to symmetric and anti-symmetric modes. The new physics found in anti-*PT*-symmetric systems can lead to new applications, i.e., symmetry-broken modes can be used for asymmetric mode switching, whereas these applications cannot be achieved using *PT*-symmetric systems. We propose a theoretical model to prove the chiral dynamics. We also perform microwave experiments to demonstrate the asymmetric mode switching for symmetry-broken modes.

## Results

### Theory of chiral dynamics in anti-*PT*-symmetric systems

**Fig. 1:**
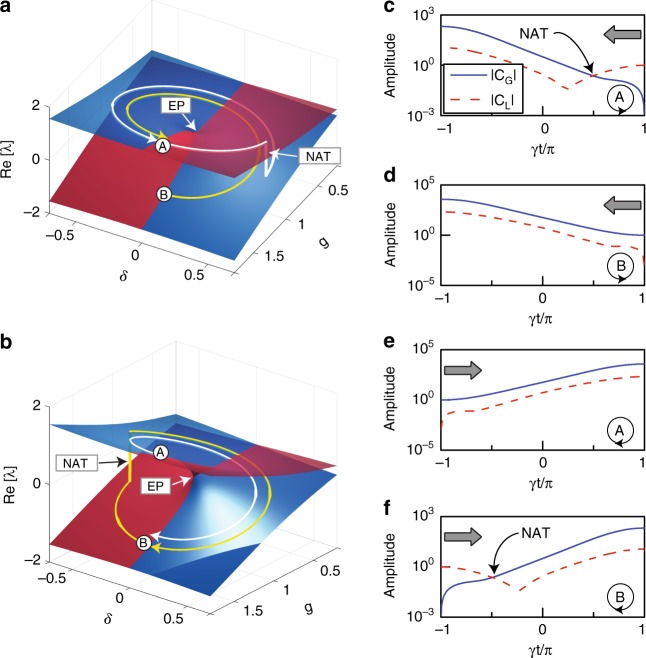
Chiral behaviour by dynamically encircling an EP in a system governed by anti-*PT*-symmetric Hamiltonian. **a** Real part of the eigenvalues as a function of *g* and *δ*. The white and yellow curves represent the trajectories for anti-clockwise loops with A and B as the initial state, respectively. **b** Same as **a** except for clockwise loops. **c–f** Calculated amplitudes of the eigenstates in the encircling process, with different encircling directions and initial states (indicated in the inset). The grey arrows mark the starting points of the loops

We start by investigating the dynamical encircling of an EP in a two-state system governed by $i\partial _t\left| {\psi \left( t \right)} \right\rangle = H\left( t \right)\left| {\psi \left( t \right)} \right\rangle$, where $\left| {\psi \left( t \right)} \right\rangle = \left[ {a\left( t \right),b\left( t \right)} \right]^T$ is the state vector at a certain time *t*. The model Hamiltonian takes the form

1 $$H\left( t \right) = \left( {\begin{array}{*{20}{c}} { - g\left( t \right) + i\delta \left( t \right)} & {i\kappa } \\ {i\kappa } & {g\left( t \right) - i\delta \left( t \right)} \end{array}} \right)$$

where *g* and *iκ* denote the amount of detuning and coupling, respectively. The system is anti-*PT*-symmetric when *δ* = 0. Without loss of generality, we set *κ* = −1 in the following analysis. We first calculate the eigenvalues *λ* of the non-Hermitian system as a function of *g* and *δ* and show the real parts in Fig. 1a. The blue sheet and red sheet correspond to the eigenstate with gain and loss, respectively. An EP can be found at *g* = 1 and *δ* = 0. We consider a loop enclosing the EP parameterized by $g\left( t \right) = 1 - \rho \cos \left( {\gamma t} \right)$ and $\delta \left( t \right) = \rho \sin \left( {\gamma t} \right)$, where the loop radius *ρ* ≤ 1 and *γ* measures the adiabaticity. When *t* = *π*/*γ* (i.e., *g* = 1 + *ρ*, *δ* = 0), the two eigenvectors are solved to be $\left| {\psi _{\mathrm{A}}} \right\rangle = \left[ {1,i\left( {\rho + 1 + \sqrt {\rho ^2 + 2\rho } } \right)} \right]^T$ and $\left| {\psi _{\mathrm{B}}} \right\rangle = \left[ {1,i\left( {\rho + 1 - \sqrt {\rho ^2 + 2\rho } } \right)} \right]^T$, indicating that the eigenstates are in the *PT*-broken phase. However, the real parts of the eigenvalues are found to bifurcate (see Fig. 1a), which is completely opposite to that in *PT*-symmetric systems, where the real parts coalesce in the *PT*-broken phase^7^. This is the key difference between *PT*-symmetric systems and anti-*PT*-symmetric systems^37,38^ and will result in different dynamics when the EP is dynamically encircled.

We consider a loop with the starting point and end point at $t_0 = - \pi /\left| \gamma \right|$ and $t_{{\mathrm{end}}} = \pi /\left| \gamma \right|$, respectively, corresponding to the *PT*-broken phase. The loop is anti-clockwise when *γ* < 0 and clockwise when *γ* > 0. We first investigate anti-clockwise loops with *ρ* = 0.5 and *γ* = −0.5. We solve the time-dependent equation numerically and extract the amplitudes of the instantaneous eigenstates at each time step, i.e., $\left| {\psi \left( t \right)} \right\rangle = C_{\mathrm{G}}\left( t \right)\left| {\psi _{\mathrm{G}}\left( t \right)} \right\rangle + C_{\mathrm{L}}\left( t \right)\left| {\psi _{\mathrm{L}}\left( t \right)} \right\rangle$, where the subscripts G and L are associated with the eigenstate on the gain sheet (Im(*λ*) > 0) and loss sheet (Im(*λ*) < 0), respectively. The obtained amplitude coefficients *C*_G_ and *C*_L_ for the process, with state A or state B being the initial state, are shown in Fig. 1c, d, respectively, and can be used to draw the trajectory of the state evolution on the energy Riemann sheets, as shown by the white and yellow curves in Fig. 1a. The trajectory is marked on the gain (loss) sheet coloured blue (red) when |*C*_G_| > |*C*_L_| (|*C*_G_| < |*C*_L_|). The yellow trajectory shows a process in which the state evolves entirely on the gain sheet (blue). The initial state B gradually transforms to state A in this stable process (see also Fig. 1d). The situation is quite different when the initial state is state A (see the white trajectory in Fig. 1a). The state at first propagates on the loss sheet (red sheet), where the state is not stable. A NAT occurs after some time, and the state jumps to the gain sheet, on which it stays for the rest of the loop. As a result, the final state is still state A, the same as the initial state because of the NAT (see also Fig. 1c). These two processes indicate that the final state for anti-clockwise loops is always state A, regardless of the initial state. We apply the same analysis to study clockwise loops. The results with *ρ* = 0.5 and *γ* = 0.5 are plotted in Fig. 1b, e, f, showing that the final state for clockwise loops is always state B, in contrast to that of anti-clockwise loops. This dynamical behaviour is called chiral dynamics, i.e., encircling the EP in different directions results in different final states that are independent of the initial state.

We give an analytical proof of the chiral dynamics using the method introduced in ref. ^26^. Inserting the expressions of *g*(*t*) and *δ*(*t*), the time-dependent Hamiltonian described by Eq. (1) can be rewritten as second-order differential equations for *a*(*t*) and *b*(*t*), e.g., $\frac{{\partial ^2a\left( t \right)}}{{\partial t^2}} + \left[ {\rho ^2e^{2i\gamma t} - \rho \left( {2 + \gamma } \right)e^{i\gamma t}} \right]a\left( t \right) = 0$, which can then be transformed into degenerate hypergeometric differential equations, and the solutions are confluent hypergeometric functions^41^. The solutions at time step *t* can be related to the initial condition by a transfer matrix, via which the final state can be proved to satisfy

2a $$\left. {b\left( {t_{{\mathrm{end}}}} \right)/a\left( {t_{{\mathrm{end}}}} \right)} \right|_{{\mathrm{ACW}}} = i\left( {\rho + 1 + \sqrt {\rho ^2 + 2\rho } } \right)$$

2b $$\left. {b\left( {t_{{\mathrm{end}}}} \right)/a\left( {t_{{\mathrm{end}}}} \right)} \right|_{{\mathrm{CW}}} = i\left( {\rho + 1 - \sqrt {\rho ^2 + 2\rho } } \right)$$

where the subscripts “ACW” and “CW” denote anti-clockwise and clockwise loops, respectively. Details of the derivation can be found in Supplementary Notes 1 and 2. The solutions indicate that regardless of the initial state, the final state is always state A for anti-clockwise loops, whereas it is state B for clockwise loops, which is exactly the chiral dynamics in anti-*PT*-symmetric systems with the starting point in the *PT*-broken phase, where the eigenstates are symmetry-broken states. This is in sharp contrast to *PT*-symmetric systems, in which the dynamics is non-chiral with a starting point in the *PT*-broken phase^34^, and only a starting point in the *PT*-symmetric phase with eigenstates being symmetric and anti-symmetric states can result in chiral dynamics^30^. The difference originates from the very different topological structures of energy Riemann surfaces of *PT*-symmetric and anti-*PT*-symmetric systems. For chiral dynamics to occur, the trajectory in the parameter space must start from a point where the two eigenstates carry the same imaginary part of the eigenvalues (see Supplementary Note 3 and Supplementary Fig. 1 for details). This is characteristic of the *PT*-symmetric phase in *PT*-symmetric systems. In contrast, it is the *PT*-broken phase that has these properties in anti-*PT*-symmetric systems. Table 1 summarizes the different dynamics in the two systems. The chiral behaviour was employed for asymmetric mode switching using the symmetric and anti-symmetric modes in *PT*-symmetric systems^30^. Based on the same principle, the symmetry-broken states can also be employed for asymmetric mode switching in anti-*PT*-symmetric systems.

**Table 1:** Comparison of the dynamical encircling of an EP in *PT*-symmetric systems and anti-*PT*-symmetric systems

System	Starting point in the *PT*-symmetric phase	Starting point in the *PT*-broken phase
*PT*-symmetric systems	Chiral dynamics^30^	Non-chiral dynamics^34^
Anti-*PT*-symmetric systems	Non-chiral dynamics [this work]	Chiral dynamics [this work]

The non-chiral dynamics in anti-*PT*-symmetric systems is discussed in Supplementary Note 5 and Supplementary Fig. 4

### Numerical demonstration of asymmetric mode switching

**Fig. 2:**
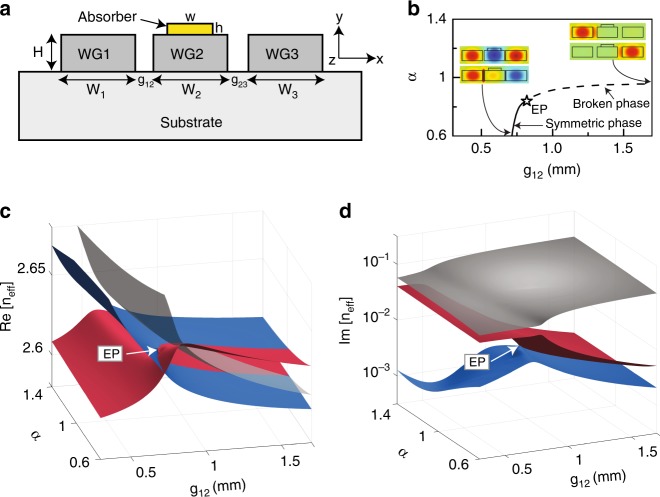
Coupled waveguide system possessing anti-*PT* symmetry. **a** Cross-sectional views of the coupled waveguides, with an absorber placed on top of waveguide-2. **b** Parameter space of the system. The star marks the EP, and the inset shows the *E*_*x*_ field distributions of the two eigenmodes on the blue and red energy sheets. **c** Real part and **d** imaginary part of the effective mode index of the system as a function of *g*_12_ and *α*

We now demonstrate the asymmetric mode switching in a realistic system consisting of three waveguides, with the cross section illustrated in Fig. 2a. The dimensions of waveguide-1 and waveguide-3 have a slight detuning (i.e., $W_1 \ne W_3$), and they are coupled via a waveguide-2 that has an absorber attached (see the yellow region). For completeness, we first demonstrate using a simple model Hamiltonian that coupling the two waveguides (i.e., waveguide-1 and waveguide-3) through a lossy waveguide-2 can effectively produce a purely imaginary value of coupling and hence anti-*PT* symmetry^38^. Consider a model Hamiltonian satisfying the equation

3 $$\left[ {\begin{array}{*{20}{c}} {\beta _1} & {{\it{\kappa }}\prime } & 0 \\ {{\it{\kappa }}\prime } & {\beta _2 + i{\it{\gamma }}\prime } & {{\it{\kappa }}\prime } \\ 0 & {{\it{\kappa }}\prime } & {\beta _3} \end{array}} \right]\left[ {\begin{array}{*{20}{c}} {\phi _1} \\ {\phi _2} \\ {\phi _3} \end{array}} \right] = E\left[ {\begin{array}{*{20}{c}} {\phi _1} \\ {\phi _2} \\ {\phi _3} \end{array}} \right]$$

where *β*_1_, $\beta _2 + i\gamma \prime$, and *β*_3_ denote the original eigenvalues of the three waveguides and $\kappa \prime$ is a real value representing the coupling between adjacent waveguides. Eliminating *ϕ*_2_, we obtain

4 $$\left\{ \begin{array}{l}\beta _1\phi _1 + \frac{{\kappa \prime ^2\phi _1 + \kappa \prime ^2\phi _3}}{{E - \beta _2 - i\gamma \prime }} = E\phi _1\\ \beta _3\phi _3 + \frac{{\kappa \prime ^2\phi _1 + \kappa \prime ^2\phi _3}}{{E - \beta _2 - i\gamma \prime }} = E\phi _3\end{array} \right.$$

For eigenvalues close to *β*_2_, Eq. (4) can be simplified as

5 $$\left[ {\begin{array}{*{20}{c}} {\beta _1 + i\frac{{\kappa \prime ^2}}{{\gamma \prime }}} & {i\frac{{\kappa \prime ^2}}{{\gamma \prime }}} \\ {i\frac{{\kappa \prime ^2}}{{\gamma \prime }}} & {\beta _3 + i\frac{{\kappa \prime ^2}}{{\gamma \prime }}} \end{array}} \right]\left[ {\begin{array}{*{20}{c}} {\phi _1} \\ {\phi _3} \end{array}} \right] = E\left[ {\begin{array}{*{20}{c}} {\phi _1} \\ {\phi _3} \end{array}} \right]$$

We note that $\beta _1 \ne \beta _3$ since there is a detuning between waveguide-1 and waveguide-3. We define $\bar \beta = \left( {\beta _1 + \beta _3} \right)/2$ and $\Delta = \left( {\beta _1 - \beta _3} \right)/2$. Then, the effective Hamiltonian describing the coupling between waveguide-1 and waveguide-3 becomes

6 $$H_{{\mathrm{eff}}} = \left[ {\begin{array}{*{20}{c}} \Delta & {i\frac{{\kappa \prime ^2}}{{\gamma \prime }}} \\ {i\frac{{\kappa \prime ^2}}{{\gamma \prime }}} & { - \Delta } \end{array}} \right]$$

after shifting the eigenvalues by a constant $\overline \beta + i\kappa \prime ^2/\gamma \prime$. We note that the eigenfields decay exponentially outside the waveguides, and as such, the coupling coefficients between neighbouring waveguides (i.e., between waveguide-1 and waveguide-2 and between waveguide-2 and waveguide-3) are real numbers (i.e., $\kappa \prime$). However, the effective coupling between waveguide-1 and waveguide-3 in the reduced two-waveguide system [going from Eq. (3) to Eq. (6)] is purely imaginary (i.e., $i\kappa \prime ^2/\gamma \prime$) and hence results in the above effective 2 × 2 Hamiltonian being anti-*PT*-symmetric. In the case of $\Delta \,>\, \kappa \prime ^2/\gamma \prime$, the eigenvalues of the effective Hamiltonian take the form $E_{1,3} = \pm \sqrt {{\Delta}^2 - \kappa ^{\prime 4}/\gamma ^{\prime 2}}$, which are real numbers and the imaginary parts coalesce. The corresponding eigenvectors are $\left| {\phi _{{\mathrm{1,3}}}} \right\rangle = \left[ {1,i\left( {\Delta \mp \sqrt {{\Delta} ^2 - \kappa \prime ^4/\gamma \prime ^2} } \right)\gamma \prime /\kappa \prime ^2} \right]^T$, indicating that the phase difference between the two waveguides in the eigenmode is π/2 and therefore the system is in the *PT*-broken phase. When $\Delta < \kappa \prime ^2/\gamma \prime$, the system is in the *PT*-symmetric phase, with the eigenvectors being $\left| {\phi _{{\mathrm{1,3}}}} \right\rangle = \left[ {1,\left( { \pm \sqrt {\kappa \prime ^4/\gamma \prime ^2 - \Delta ^2} + i\Delta } \right)\gamma \prime /\kappa \prime ^2} \right]^T$ (eigenstates will be purely symmetric and anti-symmetric states with a phase difference of 0 and π when Δ = 0), and the eigenvalues $E_{1,3} = \pm i\sqrt {\kappa \prime ^4/\gamma \prime ^2 - \Delta ^2}$ are purely imaginary values.

To demonstrate the above analysis numerically, we used COMSOL^42^ to calculate the effective mode index *n*_eff_, i.e., the eigenvalue of the waveguide system, as a function of two gap distances (i.e., *g*_12_ and *g*_23_) using structural parameters: *W*_1_ = *W*_2_ = 8 mm, *W*_3_ = 8.1 mm, *H* = 4 mm, *w* = 5 mm and *h* = 1 mm. The permittivity of the waveguides and absorber are set to 15.2 and 4 + 15*i*, respectively. The background and substrate are assumed to be air, and the frequency is 10 GHz. Although the parameter space introduced in this realistic system is different from that in the model Hamiltonian in Eq. (1), the dynamical consequence of encircling the EP is the same because the energy surfaces have the same topology. The real parts and imaginary parts of the eigenvalues are plotted in Fig. 2c, d, respectively, where we define *α* = *g*_23_/*g*_12_. The system supports three eigenmodes, which are represented by three Riemann sheets with different colours depending on the amount of loss. The grey eigenmode exhibits the highest loss, and it hardly interacts with the other two modes. We therefore focus on the red and blue Riemann sheets and find an EP located at *g*_12_ = 0.82 mm and *α* = 0.84. Figure 2b shows the parameter space. The solid and dashed curves mark, respectively, the set of points in the 2D parameter space where the real and imaginary parts of the eigenvalues coalesce. The inset shows the *E*_*x*_ field distributions of the two eigenmodes residing on the blue and red sheets. On the solid curve, the two eigenmodes are found to be symmetric and anti-symmetric (i.e., the phase difference between waveguide-1 and waveguide-3 is nearly 0 and π, respectively). In contrast, they are symmetry-broken (i.e., one mode is localized in waveguide-1, while the other one is localized in waveguide-3 with the phase difference being π/2) on the dashed curve. This indicates that the solid and dashed curves are in fact the symmetric and broken phase, respectively. It is then evident that the system is anti-*PT*-symmetric since the real/imaginary parts of the eigenvalues coalesce in the symmetric/broken phase, which is consistent with the above analysis on the effective Hamiltonian [Eq. (6)].

**Fig. 3:**
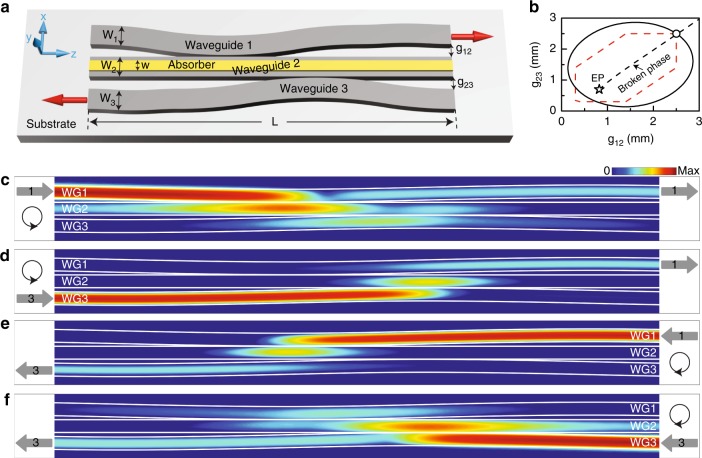
Asymmetric mode switching for symmetry-broken modes by dynamically encircling the EP in the coupled waveguide system. **a** Top view of the coupled waveguides, with two gap distances varying continuously along the waveguide direction, corresponding to the dynamical encircling of an EP in the anti-*PT*-symmetric system. The two red arrows indicate the asymmetric mode switching, i.e., power always exists in the system via waveguide-1 with left-hand side injections, whereas it exists via waveguide-3 with right-hand side injections. **b** Parameter space of the system. The circle and star mark the starting/end point and EP, respectively. The black solid curve and red dashed curve represent the trajectory of the EP encircling process in the numerical simulations and experiments, respectively; their formulas are given in the Methods section. **c–f** Numerically simulated power flow distributions in a system with different encircling directions and injections (indicated in the inset)

Figure 3b redraws the parameter space as a function of the two gap distances. We consider a black solid loop that encloses the EP, with the starting/end point at *g*_12_ = *g*_23_ = 2.5 mm, which corresponds to a point in the broken phase with eigenmodes being symmetry-broken states. A schematic diagram of a waveguide system that can mimic the designed loop is illustrated in Fig. 3a, where the two gap distances change continuously along the waveguide direction (i.e., *z*-axis). The system length *L* is set to 600 mm, and other structural parameters are kept the same as those in Fig. 2. Excitation of the initial states on the left-hand side of the system leads to state evolutions following an anti-clockwise loop, while incidence from the right-hand side leads to a clockwise loop. We calculated the wave transmission in the system, and the desired initial state at the boundary was excited by a pre-calculation of the eigenmodes using the boundary mode analysis module of COMSOL^42^. The distributions of the *z*-component power flow for anti-clockwise loops are shown in Fig. 3c, d, with the initial symmetry-broken state localized in waveguide-1 and waveguide-3, respectively. We find that regardless of the waveguide via which the power is injected, the wave always exits the system via waveguide-1. The results for clockwise loops are shown in Fig. 3e, f. In contrast, the final state is found to localize in waveguide-3 regardless of the initial state. This is a direct demonstration of the asymmetric mode switching for symmetric-broken states, which is different from that in *PT*-symmetric systems, where the asymmetric mode switching applies to the symmetric and anti-symmetric modes^30^.

**Fig. 4:**
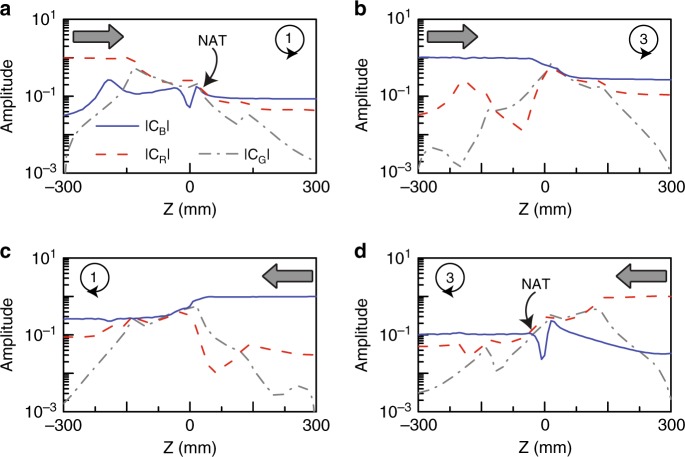
Mode amplitudes in the encircling process. **a**–**d** Extracted amplitudes of the instantaneous eigenmodes along the waveguide direction, with different encircling directions and injections (as indicated in the inset). The grey arrows mark the starting points of the loops

We further investigate the dynamics by expanding the field profiles at each *z* position as a sum of the instantaneous eigenfields, i.e., ${\mathbf{E}}_{\mathrm{t}}\left( z \right) = c_{\mathrm{B}}\left( z \right){\mathbf{E}}_{\mathrm{B}}\left( z \right) + c_{\mathrm{R}}\left( z \right){\mathbf{E}}_{\mathrm{R}}\left( z \right) + c_{\mathrm{G}}\left( z \right){\mathbf{E}}_{\mathrm{G}}\left( z \right)$, where **E**_t_ is the transverse electric field and the subscripts B, R, and G denote the instantaneous eigenmodes on the blue, red and grey Riemann sheets, respectively. The amplitude coefficients *c*_B_, *c*_R_ and *c*_G_ were determined by first constructing left eigenvectors and then performing projections of the instantaneous fields onto the left eigenfields (see Supplementary Note 4). The calculated coefficients are plotted in Fig. 4a–d, corresponding to the dynamics in Fig. 3c–f, respectively. The coefficients for the non-excited eigenmodes are not zero at the starting point, which is mainly due to reflections of a finite-length sample and numerical errors in the simulation, but they will not affect the salient features of the studied phenomenon. We find that for each encircling direction, the two processes with different initial states exhibit different dynamics. Specifically, one process is adiabatic, as the blue curve dominates the entire trajectory (see Fig. 4b, c), which enables the wave to transform from one waveguide to the other, corresponding to a state flip (see Fig. 3d, e). The other process has a NAT (at the crossing of the blue and red curves in Fig. 4a, d), and the final state is the same as the input (see Fig. 3c, f). These results well reproduce the dynamics obtained from the model Hamiltonian in Fig. 1. We also note from Fig. 4 that when the state approaches the end point, it always stays on the lower-loss blue sheet, where it is more stable. The blue sheet is not continuous in the *PT*-broken phase of anti-*PT*-symmetric systems (see Figs. 1a or 2c) such that encircling the EP in different directions leads to different final states. This is the key reason for the chiral dynamics.

### Experimental demonstration of asymmetric mode switching

**Fig. 5:**
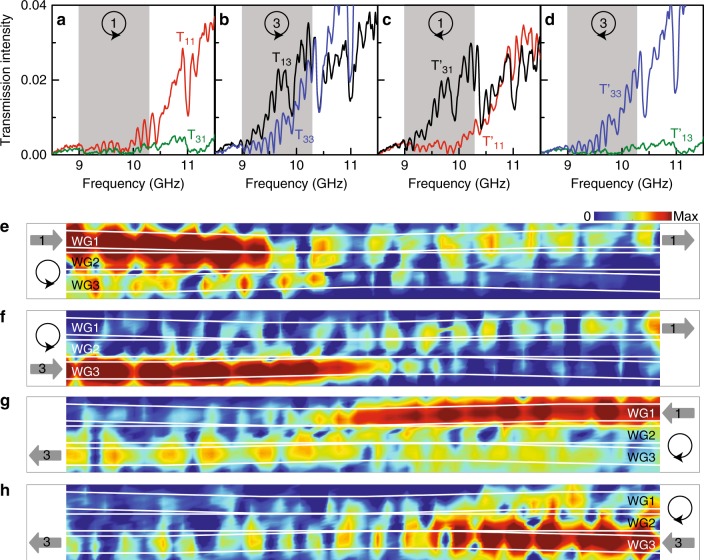
Experimental demonstration of the asymmetric mode switching for symmetry-broken modes. **a**–**d** Experimentally measured transmission spectra with different encircling directions and initial states (indicated in the inset). The grey region marks the frequency range in which the phenomenon of asymmetric mode switching can be observed. **e**–**h** The corresponding measured electric field intensity distributions on top of the waveguide system at 9.6 GHz

Microwave experiments were performed to demonstrate the asymmetric mode switching. The waveguides are made of yttrium iron garnet (YIG) with a relative permittivity of ~15.2. The trajectory of the experimental system is shown by the red dashed curve in Fig. 3b. It is slightly different from that in the numerical simulations since the system is composed of several straight YIG strips (see Supplementary Figs. 2, 3). We measured the transmission spectra for different encircling directions and initial states using an Agilent Technologies 8720ES Network Analyser. The results are shown in Fig. 5a–d, where *T*_*ij*_ ($T_{ij}^\prime$) represents the measured transmission intensity from waveguide-*j* to waveguide-*i* in an anti-clockwise (clockwise) loop. Since the system is designed to operate at 10 GHz and the location of the EP varies with the frequency, the expected phenomenon should be observable in a specific range of frequencies. These frequencies are shaded in grey in Fig. 5a–d, where we find that the transmission corresponding to anti-clockwise loops is mainly dominated by *T*_11_ (Fig. 5a) and *T*_13_ (Fig. 5b), while that of clockwise loops, by $T_{31}^\prime$ (Fig. 5c) and $T_{33}^\prime$ (Fig. 5d). This is an experimental observation of the asymmetric mode switching for symmetry-broken modes, i.e., the power always exits the system mainly via waveguide-1 in anti-clockwise loops but via waveguide-3 in clockwise loops. The oscillations of the experimental spectra are due to Fabry-Pérot resonances, as the system has a finite length of 600 mm. We note that $T_{ij} \approx T_{ji}^\prime$ since the system is reciprocal. The electric field intensity distributions were measured on top of the waveguide system (~1 mm above the surface) with the help of a stepper motor. The results at ~9.6 GHz are shown in Fig. 5e–h, corresponding to the four cases in Fig. 3c–f. We find that the experimental measurements can well reproduce the salient features of asymmetric mode switching, although the experimentally measured field distributions are not as ideal as the numerical ones, which is mainly due to their different looping trajectories (see Fig. 3b) as well as experimental imperfections. In particular, the desired eigenmodes were excited by putting an antenna close to one waveguide. In this process, the microwave radiations may also couple slightly to the undesired eigenmodes, but this will not affect the final state since it is independent of the input state. In addition, backward propagating modes and standing waves can be excited in the experiment due to the reflections at the two boundaries of the system. The chiral transmission behaviour also applies to these backward modes, which then result in some backward power flows in waveguide-3 on the left-hand side of the system (see Fig. 5e, f) and those in waveguide-1 on the right-hand side of the system (see Fig. 5g, h). This phenomenon is not observed in Fig. 3c–f due to the matched boundary conditions used in the numerical simulations.

## Conclusions

In summary, we have demonstrated new physics in anti-*PT*-symmetric systems. We showed that dynamically encircling an EP with the starting point in the *PT*-broken phase exhibits chiral dynamics due to the unique topological structure of the energy surfaces of anti-*PT*-symmetric systems. This phenomenon, unique to anti-*PT*-symmetric systems, allowed us to perform the first experiment of asymmetric mode switching for symmetry-broken modes, a functionality that is highly desirable in on-chip optical systems but cannot be realized using conventional *PT*-symmetric systems. In fact, the asymmetric mode switching of symmetric and anti-symmetric modes has recently been realized in *PT*-symmetric optical systems^30^, and the new wave-manipulation scheme proposed in this work will inspire further experiments on manipulating symmetry-broken modes in on-chip optical systems towards novel functionalities.

## Materials and methods

### Loops in the parameter space

The loop for numerical simulations (see the solid loop in Fig. 3b) takes the form

$$\left\{ \begin{array}{l}g_{12} = 1.5 - \cos \left( {2\pi z/L + \pi /4} \right)\sqrt {2\cos ^2\left( {2\pi z/L} \right) + 1.69\sin ^2\left( {2\pi z/L} \right)} \\ g_{23} = 1.5 - \sin \left( {2\pi z/L + \pi /4} \right)\sqrt {2\cos ^2\left( {2\pi z/L} \right) + 1.69\sin ^2\left( {2\pi z/L} \right)} \end{array} \right.,$$

where *L* = 600 mm and *z* = −300 ~ 300 mm. The loop for the experiments (see the dashed loop in Fig. 3b) is a polygon with vertices located at (*g*_12_, *g*_23_) = (2.5, 2.5), (1.4, 2.5), (0.3, 1.4), (0.3, 0.3), (1.4, 0.3), and (2.5, 1.4).

## Supplementary information

SUPPLEMENTARY INFORMATION for Dynamically encircling an exceptional point in anti-parity-time symmetric systems: asymmetric mode switching for symmetry-broken modes

## References

[1] Rotter I (2009). A non-Hermitian Hamilton operator and the physics of open quantum systems. J. Phys. A-Math. Theor..

[2] Moiseyev, N. *Non-Hermitian Quantum Mechanics* (Cambridge University Press, Cambridge, 2011).

[3] El-Ganainy R (2018). Non-Hermitian physics and PT symmetry. Nat. Phys..

[4] Zhao H, Feng L (2018). Parity-time symmetric photonics. National Science Review.

[5] Berry MV (2004). Physics of nonhermitian degeneracies. Czech. J. Phys..

[6] Heiss WD (2012). The physics of exceptional points. J. Phys. A-Math. Theor..

[7] Guo A (2009). Observation of *PT*-symmetry breaking in complex optical potentials. Phys. Rev. Lett..

[8] Rüter CE (2010). Observation of parity-time symmetry in optics. Nat. Phys..

[9] Benisty H (2011). Implementation of PT symmetric devices using plasmonics: principle and applications. Opt. Express.

[10] Ge L, Stone AD (2014). Parity-time symmetry breaking beyond one dimension: the role of degeneracy. Phys. Rev. X.

[11] Peng B (2014). Loss-induced suppression and revival of lasing. Science.

[12] Zhen B (2015). Spawning rings of exceptional points out of Dirac cones. Nature.

[13] Xu H (2016). Topological energy transfer in an optomechanical system with exceptional points. Nature.

[14] Hodaei H (2017). Enhanced sensitivity at higher-order exceptional points. Nature.

[15] Chen WJ (2017). Exceptional points enhance sensing in an optical microcavity. Nature.

[16] Assawaworrarit S, Yu XF, Fan SH (2017). Robust wireless power transfer using a nonlinear parity-time-symmetric circuit. Nature.

[17] Zhang XL (2017). Exceptional points and symmetry recovery in a two-state system. Phys. Rev. A.

[18] Heiss WD (2000). Repulsion of resonance states and exceptional points. Phys. Rev. E.

[19] Dembowski C (2001). Experimental observation of the topological structure of exceptional points. Phys. Rev. Lett.

[20] Gao T (2015). Observation of non-Hermitian degeneracies in a chaotic exciton-polariton billiard. Nature.

[21] Ding K (2016). Emergence, coalescence, and topological properties of multiple exceptional points and their experimental realization. Phys. Rev. X.

[22] Uzdin R, Mailybaev A, Moiseyev N (2011). On the observability and asymmetry of adiabatic state flips generated by exceptional points. J. Phys. A-Math. Theor..

[23] Berry MV, Uzdin R (2011). Slow non-Hermitian cycling: exact solutions and the Stokes phenomenon. J. Phys. A-Math. Theor..

[24] Gilary I, Mailybaev AA, Moiseyev N (2013). Time-asymmetric quantum-state-exchange mechanism. Phys. Rev. A.

[25] Milburn TJ (2015). General description of quasiadiabatic dynamical phenomena near exceptional points. Phys. Rev. A.

[26] Hassan AU (2017). Dynamically encircling exceptional points: exact evolution and polarization state conversion. Phys. Rev. Lett..

[27] Hassan AU (2017). Chiral state conversion without encircling an exceptional point. Phys. Rev. A.

[28] Graefe EM, Mailybaev AA, Moiseyev N (2013). Breakdown of adiabatic transfer of light in waveguides in the presence of absorption. Phys. Rev. A.

[29] Doppler J (2016). Dynamically encircling an exceptional point for asymmetric mode switching. Nature.

[30] Yoon JW (2018). Time-asymmetric loop around an exceptional point over the full optical communications band. Nature.

[31] Choi Y (2017). Extremely broadband, on-chip optical nonreciprocity enabled by mimicking nonlinear anti-adiabatic quantum jumps near exceptional points. Nat. Commun..

[32] Politi A (2008). Silica-on-silicon waveguide quantum circuits. Science.

[33] Piggott AY (2015). Inverse design and demonstration of a compact and broadband on-chip wavelength demultiplexer. Nat. Photon..

[34] Zhang XL (2018). Dynamically encircling exceptional points: *in situ* control of encircling loops and the role of the starting point. Phys. Rev.X.

[35] Ge L, Türeci HE (2013). Antisymmetric PT-photonic structures with balanced positive- and negative-index materials. Phys. Rev. A.

[36] Antonosyan DA, Solntsev AS, Sukhorukov AA (2015). Parity-time anti-symmetric parametric amplifier. Opt. Lett..

[37] Peng P (2016). Anti-parity-time symmetry with flying atoms. Nat. Phys..

[38] Yang F, Liu YC, You L (2017). Anti-*PT* symmetry in dissipatively coupled optical systems. Phys. Rev. A.

[39] Konotop VV, Zezyulin DA (2018). Odd-time reversal *PT* symmetry induced by an anti-*PT*-symmetric medium. Phys. Rev. Lett..

[40] Choi Y (2018). Observation of an anti-PT-symmetric exceptional point and energy-difference conserving dynamics in electrical circuit resonators. Nat. Commun..

[41] Slater, L. J. *Confluent Hypergeometric Functions.* (Cambridge University Press, Cambridge, 1960).

[42] COMSOL. *COMSOL Multiphysics® v. 4.3*. https://www.comsol.com (COMSOL AB, Stockholm, Sweden).

