# Supplementary material for: Dynamically encircling an exceptional point in anti-parity-time symmetric systems: asymmetric mode switching for symmetry-broken modes
Source: Light Sci Appl. 2019 Oct 2;8:88. doi: 10.1038/s41377-019-0200-8 (PMC6804564; doi:10.1038/s41377-019-0200-8)
Supplement: Supplementary file 1 — SUPPLEMENTARY INFORMATION for Dynamically encircling an exceptional point in anti-parity-time symmetric systems: asymmetric mode switching for symmetry-broken modes [file 41377_2019_200_MOESM1_ESM.docx]

**Supplementary Information for “Dynamically encircling an exceptional point in anti-parity-time symmetric systems: asymmetric mode switching for symmetry-broken modes”**

Xu-Lin Zhang,^1,2^ Tianshu Jiang,^2^ and C. T. Chan^2,*^

^1^State Key Laboratory of Integrated Optoelectronics, College of Electronic Science and Engineering, Jilin University, Changchun, China

*^2^Department of Physics, The Hong Kong University of Science and Technology, Clear Water Bay, Hong Kong, China*

^*^Corresponding author: [phchan@ust.hk](mailto:phchan@ust.hk)

**Supplementary Note 1**

**Analytical solutions for anti-clockwise loops (*γ* < 0)**

We consider a non-Hermitian system governed by $i\partial_{t}|\psi\left(t\right)\rangle=H\left(t\right)|\psi\left(t\right)\rangle$, where the time-dependent Hamiltonian takes the form

$H\left(t\right)=\left(\begin{matrix} -g\left(t\right)+i\delta\left(t\right) & i\kappa\\ i\kappa& g\left(t\right)-i\delta\left(t\right) \end{matrix}\right)$, (S1.1)

and $|\psi\left(t\right)\rangle=\left[a\left(t\right),b\left(t\right)\right]^{T}$ is the state vector. The system is anti-*PT*-symmetric when the detuning $\delta\left(t\right)=0$. We set *κ* to be −1 without loss of generality. The system possesses two exceptional points (EPs) located at *g* = ±1 and *δ* = 0. By introducing $g\left(t\right)=1-\rho\cos\left(\gamma t\right)$ and $\delta\left(t\right)=\rho\sin\left(\gamma t\right)$, Eq. (S1.1) can be written as

$\left\{\begin{matrix} \frac{\partial a\left(t\right)}{\partial t}+i\left(\rho e^{i\gamma t}-1\right)a\left(t\right)+b\left(t\right)=0 \\ \frac{\partial b\left(t\right)}{\partial t}-i\left(\rho e^{i\gamma t}-1\right)b\left(t\right)+a\left(t\right)=0 \end{matrix}\right.$. (S1.2)

By eliminating *b*(*t*) in Eq. (S1.2) we get

$\frac{\partial^{2}a\left(t\right)}{\partial t^{2}}+\left[\rho^{2}e^{2i\gamma t}-\rho\left(2+\gamma\right)e^{i\gamma t}\right]a\left(t\right)=0$. (S1.3)

We follow the method in Ref. [S1] to solve Eq. (S1.3). In this section, we only consider the case *γ* < 0. The case *γ* > 0 will be studied in the next section. We let $x=\gamma t$, $\alpha=-\rho^{2}/\gamma^{2}$, $\beta=\rho\left(2+\gamma\right)/\gamma^{2}$ and $z=e^{ix}$, then Eq. (S1.3) becomes

$z\frac{d^{2}a}{dz^{2}}+\frac{da}{dz}+\left(\alpha z+\beta\right)a=0$. (S1.4)

We further write $\eta=-2i\sqrt{\alpha}z$ and $a\left(\eta\right)=e^{-\eta/2}w\left(\eta\right)$, Eq. (S1.4) can then be transformed into a degenerate hypergeometric differential equation

$\eta\frac{d^{2}w}{d\eta^{2}}+\left(1-\eta\right)\frac{dw}{d\eta}-\left(-\frac{1}{\gamma}\right)w=0$. (S1.5)

The solution to Eq. (S1.5) is $w\left(\eta\right)=c_{1}F\left(-1/\gamma,1,\eta\right)+c_{2}U\left(-1/\gamma,1,\eta\right)$, and therefore

$a\left(\eta\right)=e^{-\eta/2}\left[c_{1}F\left(-\frac{1}{\gamma},1,\eta\right)+c_{2}U\left(-\frac{1}{\gamma},1,\eta\right)\right]$, (S1.6)

where *F* and *U* represent confluent hypergeometric functions of the first and second kind, respectively. The first derivative of $a\left(\eta\right)$ is

$\frac{da}{dx}=\frac{da}{d\eta}\frac{d\eta}{dx}=i\eta e^{-\eta/2}\left[-\frac{c_{1}}{2}F-\frac{c_{2}}{2}U+c_{1}\frac{dF}{d\eta}+c_{2}\frac{dU}{d\eta}\right]$.

The starting point lies in the broken phase with $t_{0}=-\pi/\left|\gamma\right|$ and thus $\eta_{0}=-2\rho/\gamma$. Considering the initial condition, we have

$\begin{matrix} a\left(t_{0}\right)=c_{1}e^{-\eta_{0}/2}F_{\eta=\eta_{0}}+c_{2}e^{-\eta_{0}/2}U_{\eta=\eta_{0}} \\ a'\left(t_{0}\right)=c_{1}i\eta_{0}e^{-\eta_{0}/2}\left(-\frac{F_{\eta=\eta_{0}}}{2}+\frac{dF_{\eta=\eta_{0}}}{d\eta}\right)+c_{2}i\eta_{0}e^{-\eta_{0}/2}\left(-\frac{U_{\eta=\eta_{0}}}{2}+\frac{dU_{\eta=\eta_{0}}}{d\eta}\right) \end{matrix}$,

which can also be written in a matrix form

$\left[\begin{matrix} a\left(t_{0}\right) \\ a'\left(t_{0}\right) \end{matrix}\right]=e^{-\eta_{0}/2}\left[\begin{matrix} F_{\eta=\eta_{0}} & U_{\eta=\eta_{0}} \\ i\eta_{0}\left(-\frac{F_{\eta=\eta_{0}}}{2}+\frac{dF_{\eta=\eta_{0}}}{d\eta}\right) & i\eta_{0}\left(-\frac{U_{\eta=\eta_{0}}}{2}+\frac{dU_{\eta=\eta_{0}}}{d\eta}\right) \end{matrix}\right]\left[\begin{matrix} c_{1} \\ c_{2} \end{matrix}\right]$.

By using the Wronskian for the hypergeometric function, the matrix can be written as

$\left[\begin{matrix} c_{1} \\ c_{2} \end{matrix}\right]=i\Gamma\left(-\frac{1}{\gamma}\right)e^{-\eta_{0}/2}\left[\begin{matrix} i\eta_{0}\left(-\frac{U_{\eta=\eta_{0}}}{2}+\frac{dU_{\eta=\eta_{0}}}{d\eta}\right) & -U_{\eta=\eta_{0}} \\ -i\eta_{0}\left(-\frac{F_{\eta=\eta_{0}}}{2}+\frac{dF_{\eta=\eta_{0}}}{d\eta}\right) & F_{\eta=\eta_{0}} \end{matrix}\right]\left[\begin{matrix} a\left(t_{0}\right) \\ a'\left(t_{0}\right) \end{matrix}\right]$.

The matrix can further be simplified by considering the differential form of the hypergeometric function,

$\begin{matrix} \frac{d}{d\eta}F\left(p_{1},p_{2},\eta\right)=\frac{p_{1}}{p_{2}}F\left(1+p_{1},1+p_{2},\eta\right) \\ \frac{d}{d\eta}U\left(p_{1},p_{2},\eta\right)=-p_{1}U\left(1+p_{1},1+p_{2},\eta\right) \end{matrix}$.

We then obtain the expression for *c*_1_ and *c*_2_,

$\left[\begin{matrix} c_{1} \\ c_{2} \end{matrix}\right]=i\Gamma\left(-\frac{1}{\gamma}\right)e^{\frac{\rho}{\gamma}}\left[\begin{matrix} \frac{i\rho}{\gamma}U_{t=-\pi/\left|\gamma\right|}^{\left(0\right)}-\frac{2i\rho}{\gamma^{2}}U_{t=-\pi/\left|\gamma\right|}^{\left(1\right)} & -U_{t=-\pi/\left|\gamma\right|}^{\left(0\right)} \\ -\frac{i\rho}{\gamma}F_{t=-\pi/\left|\gamma\right|}^{\left(0\right)}-\frac{2i\rho}{\gamma^{2}}F_{t=-\pi/\left|\gamma\right|}^{\left(1\right)} & F_{t=-\pi/\left|\gamma\right|}^{\left(0\right)} \end{matrix}\right]\left[\begin{matrix} a\left(t_{0}\right) \\ a'\left(t_{0}\right) \end{matrix}\right]$, (S1.7)

where we have defined some notations

$\begin{matrix} F^{\left(0\right)}=F\left(-\frac{1}{\gamma},1,\frac{2\rho}{\gamma}e^{i\gamma t}\right),\text{ }F^{\left(1\right)}=F\left(1-\frac{1}{\gamma},2,\frac{2\rho}{\gamma}e^{i\gamma t}\right) \\ U^{\left(0\right)}=U\left(-\frac{1}{\gamma},1,\frac{2\rho}{\gamma}e^{i\gamma t}\right),\text{ }U^{\left(1\right)}=U\left(1-\frac{1}{\gamma},2,\frac{2\rho}{\gamma}e^{i\gamma t}\right) \end{matrix}$.

We now write the solution for *a*(*t*) as

$a\left(t\right)=e^{-\frac{\rho}{\gamma}e^{i\gamma t}}\left[c_{1}F^{\left(0\right)}+c_{2}U^{\left(0\right)}\right]$, (S1.8)

and its differential form is

$\frac{da\left(t\right)}{dt}=-i\rho e^{i\gamma t-\frac{\rho}{\gamma}e^{i\gamma t}}\left[\left(c_{1}F^{\left(0\right)}+c_{2}U^{\left(0\right)}\right)+\frac{2}{\gamma}\left(c_{1}F^{\left(1\right)}-c_{2}U^{\left(1\right)}\right)\right]$. (S1.9)

The solution for *b*(*t*) can be obtained via Eq. (S1.2), Eq. (S1.8) and Eq. (S1.9)

$\begin{matrix} b\left(t\right)=-\frac{\partial a\left(t\right)}{\partial t}-i\left(\rho e^{i\gamma t}-1\right)a\left(t\right) \\ \text{ }=c_{1}\left[ie^{-\frac{\rho}{\gamma}e^{i\gamma t}}F^{\left(0\right)}+\frac{2\rho i}{\gamma}e^{i\gamma t-\frac{\rho}{\gamma}e^{i\gamma t}}F^{\left(1\right)}\right]+c_{2}\left[ie^{-\frac{\rho}{\gamma}e^{i\gamma t}}U^{\left(0\right)}-\frac{2\rho i}{\gamma}e^{i\gamma t-\frac{\rho}{\gamma}e^{i\gamma t}}U^{\left(1\right)}\right] \end{matrix}$. (S1.10)

Then we write Eq. (S1.8) and Eq. (S1.10) in a matrix form

$\left[\begin{matrix} a\left(t\right) \\ b\left(t\right) \end{matrix}\right]=e^{-\frac{\rho}{\gamma}e^{i\gamma t}}\left[\begin{matrix} F^{\left(0\right)} & U^{\left(0\right)} \\ iF^{\left(0\right)}+\frac{2\rho i}{\gamma}e^{i\gamma t}F^{\left(1\right)} & iU^{\left(0\right)}-\frac{2\rho i}{\gamma}e^{i\gamma t}U^{\left(1\right)} \end{matrix}\right]\left[\begin{matrix} c_{1} \\ c_{2} \end{matrix}\right]$. (S1.11)

By using Eq. (S1.2), we find

$\left[\begin{matrix} a\left(t_{0}\right) \\ a'\left(t_{0}\right) \end{matrix}\right]=\left[\begin{matrix} 1 & 0 \\ \frac{i\left(1+\rho\right)}{\gamma} & -\frac{1}{\gamma} \end{matrix}\right]\left[\begin{matrix} a\left(t_{0}\right) \\ b\left(t_{0}\right) \end{matrix}\right]$. (S1.12)

Combining Eq. (S1.7), Eq. (S1.11) and Eq. (S1.12), we obtain

$\left[\begin{matrix} a\left(t\right) \\ b\left(t\right) \end{matrix}\right]=\sigma\left(t\right)M_{1}\left(t\right)M_{2}M_{3}\left[\begin{matrix} a\left(t_{0}\right) \\ b\left(t_{0}\right) \end{matrix}\right]$, (S1.13)

where $\sigma\left(t\right)=i\Gamma\left(-1/\gamma\right)e^{\frac{\rho}{\gamma}\left(1-e^{i\gamma t}\right)}$ and

$\begin{matrix} \text{ }M_{1}\left(t\right)=\left[\begin{matrix} F^{\left(0\right)} & U^{\left(0\right)} \\ iF^{\left(0\right)}+\frac{2\rho i}{\gamma}e^{i\gamma t}F^{\left(1\right)} & iU^{\left(0\right)}-\frac{2\rho i}{\gamma}e^{i\gamma t}U^{\left(1\right)} \end{matrix}\right], \\ M_{2}=\left[\begin{matrix} \frac{i\rho}{\gamma}U_{t=-\pi/\left|\gamma\right|}^{\left(0\right)}-\frac{2i\rho}{\gamma^{2}}U_{t=-\pi/\left|\gamma\right|}^{\left(1\right)} & -U_{t=-\pi/\left|\gamma\right|}^{\left(0\right)} \\ -\frac{i\rho}{\gamma}F_{t=-\pi/\left|\gamma\right|}^{\left(0\right)}-\frac{2i\rho}{\gamma^{2}}F_{t=-\pi/\left|\gamma\right|}^{\left(1\right)} & F_{t=-\pi/\left|\gamma\right|}^{\left(0\right)} \end{matrix}\right],\text{ }M_{3}=\left[\begin{matrix} 1 & 0 \\ \frac{i\left(1+\rho\right)}{\gamma} & -\frac{1}{\gamma} \end{matrix}\right] \end{matrix}$. (S1.14)

Now we consider the final state $\left[a_{\text{end}},b_{\text{end}}\right]^{T}$ at $t_{\text{end}}=\pi/\left|\gamma\right|$. From Eq. (S1.13) we find

$\frac{b_{\text{end}}}{a_{\text{end}}}=\frac{m_{21}a\left(t_{0}\right)+m_{22}b\left(t_{0}\right)}{m_{11}a\left(t_{0}\right)+m_{12}b\left(t_{0}\right)}$, (S1.15)

where we have defined a matrix

$M\left(t=\pi/\left|\gamma\right|\right)=M_{1}\left(t=\pi/\left|\gamma\right|\right)M_{2}M_{3}=\left[\begin{matrix} m_{11} & m_{12} \\ m_{21} & m_{22} \end{matrix}\right]$.

To find the solution to Eq. (S1.15), we first find the analytical expression of the matrix elements which are given by

$\begin{matrix} m_{11}=\frac{i}{\gamma}\left(U_{t=\pi/\left|\gamma\right|}^{\left(0\right)}F_{t=-\pi/\left|\gamma\right|}^{\left(0\right)}-F_{t=\pi/\left|\gamma\right|}^{\left(0\right)}U_{t=-\pi/\left|\gamma\right|}^{\left(0\right)}\right)-\frac{2i\rho}{\gamma^{2}}\left(F_{t=\pi/\left|\gamma\right|}^{\left(0\right)}U_{t=-\pi/\left|\gamma\right|}^{\left(1\right)}+U_{t=\pi/\left|\gamma\right|}^{\left(0\right)}F_{t=-\pi/\left|\gamma\right|}^{\left(1\right)}\right) \\ m_{12}=-\frac{1}{\gamma}\left(U_{t=\pi/\left|\gamma\right|}^{\left(0\right)}F_{t=-\pi/\left|\gamma\right|}^{\left(0\right)}-F_{t=\pi/\left|\gamma\right|}^{\left(0\right)}U_{t=-\pi/\left|\gamma\right|}^{\left(0\right)}\right) \\ m_{21}=\frac{2\rho}{\gamma^{2}}\left(F_{t=\pi/\left|\gamma\right|}^{\left(0\right)}U_{t=-\pi/\left|\gamma\right|}^{\left(1\right)}+U_{t=\pi/\left|\gamma\right|}^{\left(0\right)}F_{t=-\pi/\left|\gamma\right|}^{\left(1\right)}\right)-\frac{4\rho^{2}}{\gamma^{3}}\left(F_{t=\pi/\left|\gamma\right|}^{\left(1\right)}U_{t=-\pi/\left|\gamma\right|}^{\left(1\right)}-U_{t=\pi/\left|\gamma\right|}^{\left(1\right)}F_{t=-\pi/\left|\gamma\right|}^{\left(1\right)}\right) \\ \text{ }+\frac{1}{\gamma}\left(F_{t=\pi/\left|\gamma\right|}^{\left(0\right)}U_{t=-\pi/\left|\gamma\right|}^{\left(0\right)}-U_{t=\pi/\left|\gamma\right|}^{\left(0\right)}F_{t=-\pi/\left|\gamma\right|}^{\left(0\right)}\right)-\frac{2\rho}{\gamma^{2}}\left(F_{t=\pi/\left|\gamma\right|}^{\left(1\right)}U_{t=-\pi/\left|\gamma\right|}^{\left(0\right)}+U_{t=\pi/\left|\gamma\right|}^{\left(1\right)}F_{t=-\pi/\left|\gamma\right|}^{\left(0\right)}\right) \\ m_{22}=\frac{i}{\gamma}\left(F_{t=\pi/\left|\gamma\right|}^{\left(0\right)}U_{t=-\pi/\left|\gamma\right|}^{\left(0\right)}-U_{t=\pi/\left|\gamma\right|}^{\left(0\right)}F_{t=-\pi/\left|\gamma\right|}^{\left(0\right)}\right)-\frac{2i\rho}{\gamma^{2}}\left(F_{t=\pi/\left|\gamma\right|}^{\left(1\right)}U_{t=-\pi/\left|\gamma\right|}^{\left(0\right)}+U_{t=\pi/\left|\gamma\right|}^{\left(1\right)}F_{t=-\pi/\left|\gamma\right|}^{\left(0\right)}\right) \end{matrix}$.

The principal value of $U\left(p_{1},p_{2},\eta\right)$ lies in $-\pi<\arg\left(\eta\right)<\pi$. It is easy to find that $U_{t=-\pi/\left|\gamma\right|}^{\left(0\right)}$ and $U_{t=-\pi/\left|\gamma\right|}^{\left(1\right)}$ are within this range whereas $U_{t=\pi/\left|\gamma\right|}^{\left(0\right)}$ and $U_{t=\pi/\left|\gamma\right|}^{\left(1\right)}$ are out of the range since *γ* < 0. We can use a connection formula (see Eq. (2.2.20) in Ref. [S2])

$U_{t=-\pi/\left|\gamma\right|}^{\left(0\right)}=-\frac{2\pi i}{\Gamma\left(-1/\gamma\right)}F_{t=\pi/\left|\gamma\right|}^{\left(0\right)}+U_{t=\pi/\left|\gamma\right|}^{\left(0\right)},\text{ }U_{t=-\pi/\left|\gamma\right|}^{\left(1\right)}=\frac{2\pi i}{\Gamma\left(-1/\gamma\right)}F_{t=\pi/\left|\gamma\right|}^{\left(1\right)}+U_{t=\pi/\left|\gamma\right|}^{\left(1\right)}$.

We also have $F_{t=-\pi/\left|\gamma\right|}^{\left(0\right)}=F_{t=\pi/\left|\gamma\right|}^{\left(0\right)}$ and $F_{t=-\pi/\left|\gamma\right|}^{\left(1\right)}=F_{t=\pi/\left|\gamma\right|}^{\left(1\right)}$ so that the expression of the matrix elements can further be simplified using the terms related to $t=\pi/\left|\gamma\right|$ only

$\begin{matrix} m_{11}=-\frac{2\pi}{\gamma\Gamma\left(-1/\gamma\right)}F^{\left(0\right)}F^{\left(0\right)}+\frac{4\pi\rho}{\gamma^{2}\Gamma\left(-1/\gamma\right)}F^{\left(0\right)}F^{\left(1\right)} \\ m_{12}=-\frac{2\pi i}{\gamma\Gamma\left(-1/\gamma\right)}F^{\left(0\right)}F^{\left(0\right)} \\ m_{21}=-\frac{2\pi i}{\gamma\Gamma\left(-1/\gamma\right)}F^{\left(0\right)}F^{\left(0\right)}+\frac{8\pi\rho i}{\gamma^{2}\Gamma\left(-1/\gamma\right)}F^{\left(0\right)}F^{\left(1\right)}-\frac{8\pi\rho^{2}i}{\gamma^{3}\Gamma\left(-1/\gamma\right)}F^{\left(1\right)}F^{\left(1\right)} \\ m_{22}=\frac{2\pi}{\gamma\Gamma\left(-1/\gamma\right)}F^{\left(0\right)}F^{\left(0\right)}-\frac{4\pi\rho}{\gamma^{2}\Gamma\left(-1/\gamma\right)}F^{\left(0\right)}F^{\left(1\right)} \end{matrix}$, (S1.16)

where we have omitted the subscript $t=\pi/\left|\gamma\right|$. We note that in the limit $\gamma\to0$ (adiabatic limit), the values of $\frac{2i\rho}{\gamma^{2}}F^{\left(0\right)}U^{\left(1\right)}$ and $\frac{2i\rho}{\gamma^{2}}F^{\left(1\right)}U^{\left(0\right)}$ are considerably smaller than other terms so that we have also omitted them in Eq. (S1.16). To further simplify these expressions of the matrix elements, we need find the analytical form of $F^{\left(0\right)}$ and $F^{\left(1\right)}$. We use the asymptotic expansion of $F\left(p_{1},p_{2},\eta\right)$ when *p*_1_ is large (see Eq. (4.4.14) in Ref. [S2])

$F\left(p_{1},p_{2},\eta\right)\approx\Gamma\left(p_{2}\right)e^{\frac{\eta}{2}}\left(k\eta\right)^{\frac{1}{4}-\frac{p_{2}}{2}}\pi^{-\frac{1}{2}}\cos\left(2\sqrt{k\eta}-\frac{p_{2}\pi}{2}+\frac{\pi}{4}\right)$,

where $k=p_{2}/2-p_{1}$. Then we have

$\begin{matrix} F^{\left(0\right)}=F\left(-\frac{1}{\gamma},1,-\frac{2\rho}{\gamma}\right)\approx e^{-\frac{\rho}{\gamma}}\left(-\frac{\rho}{\gamma}-\frac{2\rho}{\gamma^{2}}\right)^{-\frac{1}{4}}\pi^{-\frac{1}{2}}\cos\left(2\sqrt{-\frac{\rho}{\gamma}-\frac{2\rho}{\gamma^{2}}}-\frac{\pi}{4}\right) \\ F^{\left(1\right)}=F\left(1-\frac{1}{\gamma},2,-\frac{2\rho}{\gamma}\right)\approx e^{-\frac{\rho}{\gamma}}\left(-\frac{2\rho}{\gamma^{2}}\right)^{-\frac{3}{4}}\pi^{-\frac{1}{2}}\cos\left(2\sqrt{-\frac{2\rho}{\gamma^{2}}}-\frac{3\pi}{4}\right) \end{matrix}$,

which implies

$\frac{F^{\left(1\right)}}{F^{\left(0\right)}}\approx\frac{i\gamma}{\sqrt{2\rho}}\frac{\cos\left(2\sqrt{-\frac{2\rho}{\gamma^{2}}}-\frac{3\pi}{4}\right)}{\cos\left(2\sqrt{-\frac{\rho}{\gamma}-\frac{2\rho}{\gamma^{2}}}-\frac{\pi}{4}\right)}$. (S1.17)

Furthermore, the two cosine functions can be expressed as

$\begin{matrix} \cos\left(2\sqrt{-\frac{2\rho}{\gamma^{2}}}-\frac{3\pi}{4}\right)=-\frac{\sqrt{2}}{2}\left(\cos\left(\frac{-i2\sqrt{2\rho}}{\gamma}\right)-\sin\left(\frac{-i2\sqrt{2\rho}}{\gamma}\right)\right)\approx-\frac{1}{2\sqrt{2}}\left(1-i\right)e^{-\frac{2\sqrt{2\rho}}{\gamma}} \\ \cos\left(2\sqrt{-\frac{\rho}{\gamma}-\frac{2\rho}{\gamma^{2}}}-\frac{\pi}{4}\right)\approx\cos\left(\frac{-i2\sqrt{2\rho}}{\gamma}-i\sqrt{\frac{\rho}{2}}-\frac{\pi}{4}\right) \\ =\frac{\sqrt{2}}{2}\left(\cos\left(-\frac{i2\sqrt{2\rho}}{\gamma}-i\sqrt{\frac{\rho}{2}}\right)+\sin\left(-\frac{i2\sqrt{2\rho}}{\gamma}-i\sqrt{\frac{\rho}{2}}\right)\right)\approx\frac{1}{2\sqrt{2}}\left(1+i\right)e^{-\frac{2\sqrt{2\rho}}{\gamma}-\sqrt{\frac{\rho}{2}}} \end{matrix}$,

where we have used the approximation and when is large. Using these approximate expressions, Eq. (S1.17) reads

$\frac{F^{\left(1\right)}}{F^{\left(0\right)}}\approx\frac{-\gamma}{\sqrt{2\rho}}e^{\sqrt{\frac{\rho}{2}}}\approx\frac{-\gamma}{\sqrt{2\rho}}\left(1+\sqrt{\frac{\rho}{2}}+\frac{\rho}{4}\right)=-\gamma\left(\frac{1}{\sqrt{2\rho}}+\frac{1}{2}+\frac{\sqrt{2\rho}}{8}\right)$, (S1.18)

where a Taylor expansion for $e^{\sqrt{\frac{\rho}{2}}}$ is used for $\rho\leq1$. Inserting Eq. (S1.18) into Eq. (S1.16), we have

$\begin{matrix} \frac{m_{21}}{m_{11}}=\frac{i\gamma^{2}F^{\left(0\right)}F^{\left(0\right)}-4\rho\gamma iF^{\left(0\right)}F^{\left(1\right)}+4\rho^{2}iF^{\left(1\right)}F^{\left(1\right)}}{\gamma^{2}F^{\left(0\right)}F^{\left(0\right)}-2\rho\gamma F^{\left(0\right)}F^{\left(1\right)}} \\ \text{ }=\frac{i\gamma^{2}-4\rho\gamma i\frac{F^{\left(1\right)}}{F^{\left(0\right)}}+4\rho^{2}i\left(\frac{F^{\left(1\right)}}{F^{\left(0\right)}}\right)^{2}}{\gamma^{2}-2\rho\gamma\frac{F^{\left(1\right)}}{F^{\left(0\right)}}}=i\frac{1+2\sqrt{2\rho}e^{\sqrt{\frac{\rho}{2}}}+2\rho e^{2\sqrt{\frac{\rho}{2}}}}{1+\sqrt{2\rho}e^{\sqrt{\frac{\rho}{2}}}} \\ \text{ }=i\left(1+\sqrt{2\rho}e^{\sqrt{\frac{\rho}{2}}}\right)=i\left(1+\rho+\sqrt{\rho^{2}+2\rho}\right) \end{matrix}$,

and

$\begin{matrix} \frac{m_{22}}{m_{12}}=\frac{-\gamma F^{\left(0\right)}F^{\left(0\right)}\text{+}2\rho F^{\left(0\right)}F^{\left(1\right)}}{i\gamma F^{\left(0\right)}F^{\left(0\right)}}=\frac{-\gamma\text{+}2\rho\frac{F^{\left(1\right)}}{F^{\left(0\right)}}}{i\gamma} \\ \text{ }=i\left(1+\sqrt{2\rho}e^{\sqrt{\frac{\rho}{2}}}\right)=i\left(1+\rho+\sqrt{\rho^{2}+2\rho}\right) \end{matrix}$,

which means

$\frac{m_{21}}{m_{11}}=\frac{m_{22}}{m_{12}}=i\left(1+\rho+\sqrt{\rho^{2}+2\rho}\right)$. (S1.19)

Combining Eq. (S1.15) and Eq. (S1.19), we finally obtain an analytical expression of the final state

$\frac{b_{\text{end}}}{a_{\text{end}}}=\frac{m_{21}a\left(t_{0}\right)+m_{22}b\left(t_{0}\right)}{m_{11}a\left(t_{0}\right)+m_{12}b\left(t_{0}\right)}=i\left(1+\rho+\sqrt{\rho^{2}+2\rho}\right)$, (S1.20)

which indicates that the final state is independent of the initial state. At the starting/end point ($t=\pm\pi/\left|\gamma\right|$), the two eigenstates are $|\psi_{\text{A}}\rangle=\left[1,i\left(\rho+1+\sqrt{\rho^{2}+2\rho}\right)\right]^{T}$and $|\psi_{\text{B}}\rangle=\left[1,i\left(\rho+1-\sqrt{\rho^{2}+2\rho}\right)\right]^{T}$. Therefore, the final state of anti-clockwise loops (*γ* < 0) is always $|\psi_{\text{A}}\rangle$ regardless of the input state.

**Supplementary Note 2**

**Analytical solutions for clockwise loops (*γ* > 0) and chiral dynamics**

We have derived the analytical solutions of anti-clockwise loops in the last section. In this section, we show that the results for clockwise loops can be obtained by doing a transformation. We reconsider the coupled equations for anti-clockwise loops which are

$\left\{\begin{matrix} \frac{\partial a_{\text{CCW}}}{\partial t}+i\left(\rho e^{+i\gamma t}-1\right)a_{\text{CCW}}+b_{\text{CCW}}=0 \\ \frac{\partial b_{\text{CCW}}}{\partial t}-i\left(\rho e^{+i\gamma t}-1\right)b_{\text{CCW}}+a_{\text{CCW}}=0 \end{matrix}\right.$, (S2.1)

where the subscript ACW indicates an anti-clockwise loop. Now we consider a clockwise loop by changing $\gamma\to-\gamma$ and we have

$\left\{\begin{matrix} \frac{\partial a_{\text{CW}}}{\partial t}+i\left(\rho e^{-i\gamma t}-1\right)a_{\text{CW}}+b_{\text{CW}}=0 \\ \frac{\partial b_{\text{CW}}}{\partial t}-i\left(\rho e^{-i\gamma t}-1\right)b_{\text{CW}}+a_{\text{CW}}=0 \end{matrix}\right.$. (S2.2)

By taking a complex conjugate of Eq. (S2.2) we have

$\left\{\begin{matrix} \frac{\partial a_{\text{CW}}^{*}}{\partial t}-i\left(\rho e^{+i\gamma t}-1\right)a_{\text{CW}}^{*}+b_{\text{CW}}^{*}=0 \\ \frac{\partial b_{\text{CW}}^{*}}{\partial t}+i\left(\rho e^{+i\gamma t}-1\right)b_{\text{CW}}^{*}+a_{\text{CW}}^{*}=0 \end{matrix}\right.$. (S2.3)

By comparing Eq. (S2.3) with Eq. (S2.1), we find that $\left(a_{\text{CW}}^{*},b_{\text{CW}}^{*}\right)$ have the same dynamics as $\left(b_{\text{CCW}},a_{\text{CCW}}\right)$. The final state for clockwise loops can be obtained by

$\left.\frac{b_{\text{end}}}{a_{\text{end}}}\right|_{\text{CW}}=\left(\left.\frac{a_{\text{end}}}{b_{\text{end}}}\right|_{\text{CCW}}\right)^{*}=\left(\frac{1}{i\left(1+\rho+\sqrt{\rho^{2}+2\rho}\right)}\right)^{*}=i\left(1+\rho-\sqrt{\rho^{2}+2\rho}\right)$, (S2.4)

which indicates that the final state of clockwise loops (*γ* > 0) is always $|\psi_{\text{B}}\rangle$ regardless of the initial state. This is chiral dynamics, i.e., anti-clockwise loops result in the state $|\psi_{\text{A}}\rangle$ while clockwise loops the state $|\psi_{\text{B}}\rangle$. In conventional *PT*-symmetric systems, such chiral dynamics are found with the starting/end point in the *PT*-symmetric phase. Here we show that in anti-*PT*-symmetric systems, the states in the *PT*-broken phase exhibit the chiral dynamics.

**Supplementary Note 3**

**Origin of the chiral dynamics**

We show in this part the origin of the chiral dynamics in the process of dynamically encircling EPs. Supplementary Fig. 1 shows a typical energy surface of a two-state non-Hermitian system, where *λ* denotes the eigenvalue of the system. The star marks the EP and the dashed line in Supplementary Fig. 1b corresponds to the region where the two eigenmodes share the same gain and loss. We consider a loop with the starting and end point in this region and the two eigenmodes at the starting and end point are represented by the two circles. When the system is *PT*-symmetric, the states on the dashed line are in the *PT*-symmetric phase, on which mode 1 is a symmetric mode while mode 2 is an antisymmetric mode. The blue sheet has a lower loss than the red sheet. One important rule in the process is that the state is stable when it evolves on the lowest loss sheet. On the contrary, it is not stable when it is on the higher loss sheet and non-adiabatic transition will occur after some delay time. This rule has been demonstrated in many references (e.g., Refs. [S3]-[S6]). Based on this rule, we can find from the energy surface that as long as the system (i.e., the waveguide) is long enough (that means each non-adiabatic transition has enough time to occur), the state must end up on the lower loss sheet (i.e., the blue sheet) when it approaches the end point. Therefore, the final state is mode 1 for clockwise (CW) loops and mode 2 for anti-clockwise (ACW) loops, due to the fact that the blue sheet is not continuous there. This is the origin of the chiral dynamics. If the starting point lies somewhere else, e.g., *PT*-broken phase, the blue sheet is continuous and the dynamics is found to be non-chiral (see Ref. [S7]). This is why the chiral dynamics requires that the two eigenmodes share the same loss at the starting and end point of the loop. This statement also applies to anti-*PT*-symmetric systems as demonstrated in this work. The topological structure of anti-*PT*-symmetric systems is opposite to that of *PT*-symmetric systems, i.e., the two eigenmodes share the same loss in the *PT*-broken phase. As a result, the symmetry-broken modes there (i.e., mode 1 and mode 2) can be used for asymmetric mode switching, which cannot be realized in *PT*-symmetric systems due to their different topological structures.

**Supplementary Note 4**

**Extracting the amplitudes of the eigenmodes in waveguide systems**

We show the method to extract the amplitudes of the eigenmodes in the three-waveguide system. The corresponding transverse electric and magnetic eigenfields are denoted by $\mathbf{E}_{1}^{R}$, $\mathbf{E}_{2}^{R}$, $\mathbf{E}_{3}^{R}$ and $\mathbf{H}_{1}^{R}$, $\mathbf{H}_{2}^{R}$, $\mathbf{H}_{3}^{R}$, where the superscript *R* indicates that they are right eigenvectors. We first construct their left eigenvectors defined as $\mathbf{E}_{1}^{L}$, $\mathbf{E}_{2}^{L}$, $\mathbf{E}_{3}^{L}$ and $\mathbf{H}_{1}^{L}$, $\mathbf{H}_{2}^{L}$, $\mathbf{H}_{3}^{L}$. We take eigenmode 1 for example and assume the left eigenvectors take the form

$\left\{\begin{matrix} \mathbf{E}_{1}^{L}=\mathbf{E}_{1}^{R}-\alpha_{1}\mathbf{E}_{2}^{R}-\beta_{1}\mathbf{E}_{3}^{R} \\ \mathbf{H}_{1}^{L}=\mathbf{H}_{1}^{R}-\alpha_{1}\mathbf{H}_{2}^{R}-\beta_{1}\mathbf{H}_{3}^{R} \end{matrix}\right.$, (S3.1)

where *α*_1_ and *β*_1_ are coefficients to be determined. The right and left eigenvectors should satisfy the orthogonal relation so that we have

, (S3.2)

where the integration is performed over the entire waveguide cross section *S*. For the simplicity of representation, we define

, (S3.3)

where *m*,*n* = 1,2,3. Inserting Eq. (S3.1) into Eq. (S3.2), we have

$\left\{\begin{matrix} f_{1,2}-\alpha_{1}f_{2,2}-\beta_{1}f_{3,2}=0 \\ f_{1,3}-\alpha_{1}f_{2,3}-\beta_{1}f_{3,3}=0 \end{matrix}\right.$. (S3.4)

Then we can solve Eq. (S3.4) to obtain the coefficients

$\left\{\begin{matrix} \alpha_{1}=\frac{f_{1,2}f_{3,3}-f_{1,3}f_{3,2}}{f_{3,3}f_{2,2}-f_{3,2}f_{2,3}} \\ \beta_{1}=\frac{f_{1,3}f_{2,2}-f_{1,2}f_{2,3}}{f_{3,3}f_{2,2}-f_{3,2}f_{2,3}} \end{matrix}\right.$. (S3.5)

We now get the left eigenvectors. Consider the transverse field distributions as a linear combination of the right eigenfields:

$\left\{\begin{matrix} \mathbf{E}_{t}\left(x,y\right)=c_{1}\mathbf{E}_{1}^{R}\left(x,y\right)+c_{2}\mathbf{E}_{2}^{R}\left(x,y\right)+c_{3}\mathbf{E}_{3}^{R}\left(x,y\right) \\ \mathbf{H}_{t}\left(x,y\right)=c_{1}\mathbf{H}_{1}^{R}\left(x,y\right)+c_{2}\mathbf{H}_{2}^{R}\left(x,y\right)+c_{3}\mathbf{H}_{3}^{R}\left(x,y\right) \end{matrix}\right.$. (S3.6)

We can then solve the amplitude coefficients by projecting the transverse field distribution onto the left eigenvectors, e.g.,

. (S3.7)

Using this method, we extract the amplitude coefficients of instantaneous eigenmodes in the waveguide system and the results are shown in Fig. 4 of the main text.

**Supplementary Note 5**

**Non-chiral dynamics with the starting point in *PT*-symmetric phase**

We now discuss the dynamical encircling of an EP in anti-*PT*-symmetric systems with the starting point in the *PT*-symmetric phase. The starting point and end point are fixed at and , respectively. The eigenvectors at the starting/end point are and , where . The corresponding eigenvalues are and . It is easy to see that state C stays on the loss sheet whereas state D on the gain sheet. We solve the encircling process and extract the amplitude coefficients of the instantaneous eigenstates with *ρ* = 1 and *γ* = ±0.1. The results are shown in Supplementary Fig. 4 for different encircling directions and initial states as indicated in each inset. The vertical dashed line in each figure marks the branch cut, which connects the gain sheet and loss sheet, meaning that the state can leave one sheet and get on to the other one via the branch cut. We find for each encircling direction, the two processes have different dynamics. The one with state C (loss state) as input has two non-adiabatic transitions (NATs) while the other one with state D (gain sheet) as the initial state has only one NAT. Although the number of NATs occurred is different, the final state is always state D which is on the gain sheet, even independent of the encircling directions. The dynamics is non-chiral, i.e., encircling the EP in different directions result in the same final state. This is in contrast to the chiral dynamics found in the main text when the starting point lies in the *PT*-broken phase. The non-chiral dynamics can also be understood from the fact that the state would be on the gain sheet when it approaches the end point. From the Riemann sheet shown in Fig. 1a of the main text, we see that the blue (gain) sheet in the *PT*-symmetric phase in continuous. As a result, anti-clockwise and clockwise loops have the same output state, resulting in a non-chiral behaviour.


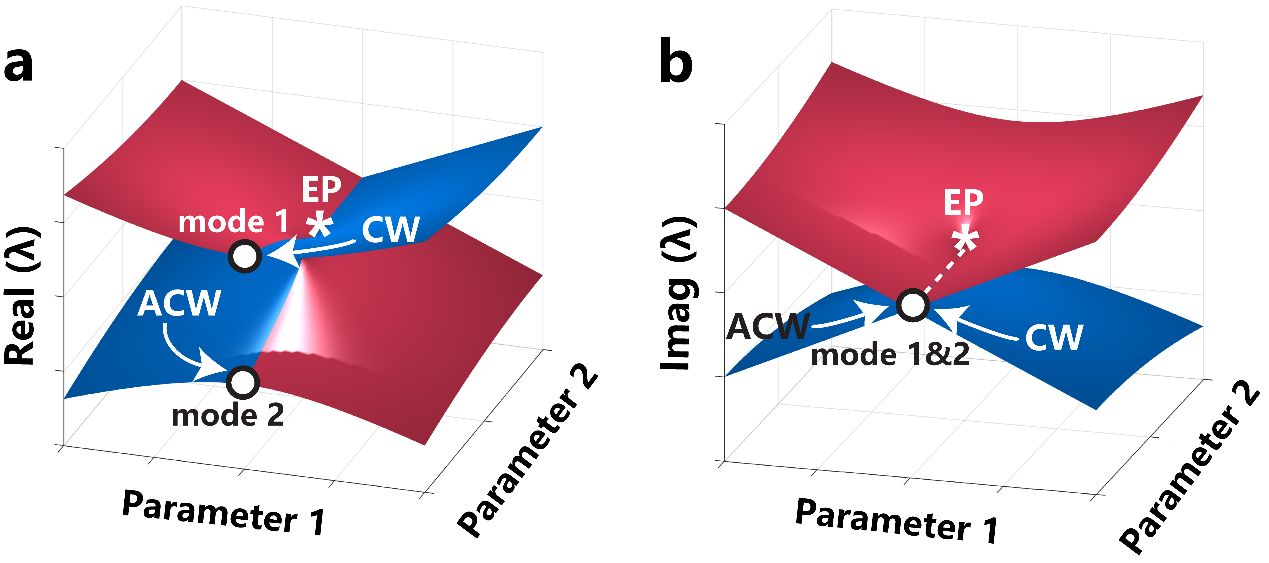


**Supplementary Figure 1. Origin of the chiral dynamics.** **a-b** Real part and imaginary part of the energy Riemann surface of a two-state non-Hermitian system.


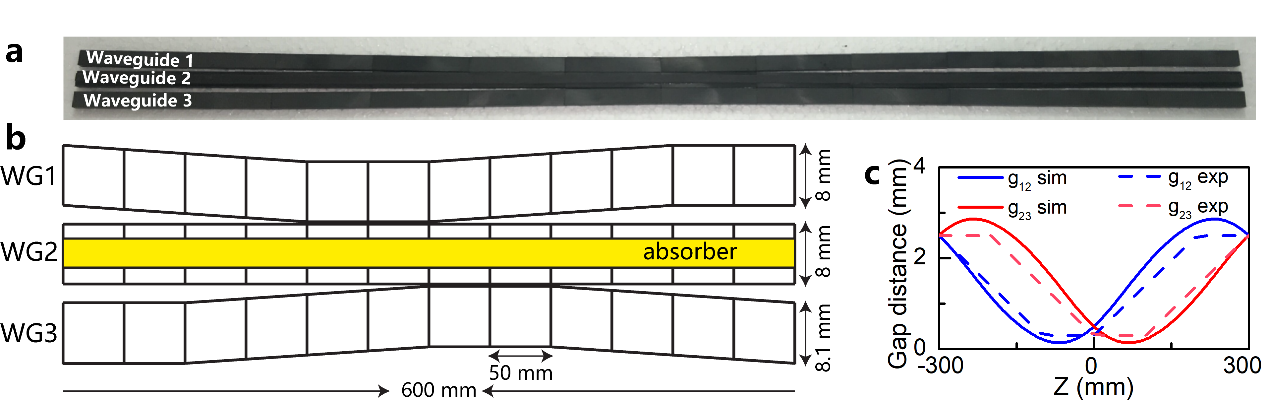


**Supplementary Figure 2. Details of the experimental system.** **a** Photograph of the three YIG waveguides. **b** Schematic diagram of the system in experiment. **c** The variation of the two gap distances used for numerical simulations (solid lines) and experiments (dashed lines). The waveguides used in experiment are made of yttrium iron garnet (YIG). We choose YIG because it has a high relative permittivity (~15.2). It is a ferromagnetic material but we do not use its ferromagnetic properties. Each waveguide consists of 12 straight YIG strips (see **b**). Each YIG strip has a height of 4 mm, width of 8 mm (for waveguide-1 and waveguide-2) or 8.1 mm (for waveguide-3) and length of ~50 mm. Since there are some corners in waveguide-1 and waveuide-3, we polished the edge of the corresponding YIG stripe slightly using a hand polisher in order align the two YIG stripes around each corner. The two gap distances *g*_12_ and *g*_23_ are varying continuously along the waveguiding direction, with the corresponding values plotted by the two dashed lines in **c**. For comparison, the values of *g*_12_ and *g*_23_ used in numerical simulations (see Fig. 3 of the main text) are also plotted by the solid lines. Their trajectories are slightly different and shown in Fig. 3b of the main text.


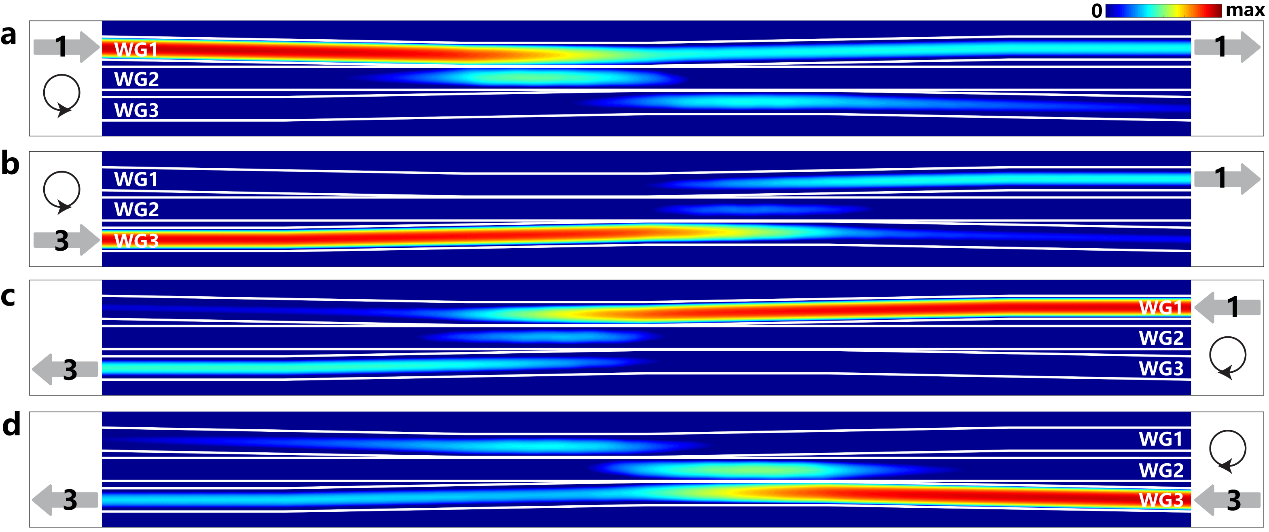


**Supplementary Figure 3. Numerical simulations of the experimental system.** **a-d** Numerically simulated power flow distributions of the experimental system with different encircling directions and injections (indicated in the inset). The phenomenon of asymmetric mode switching is very clear.


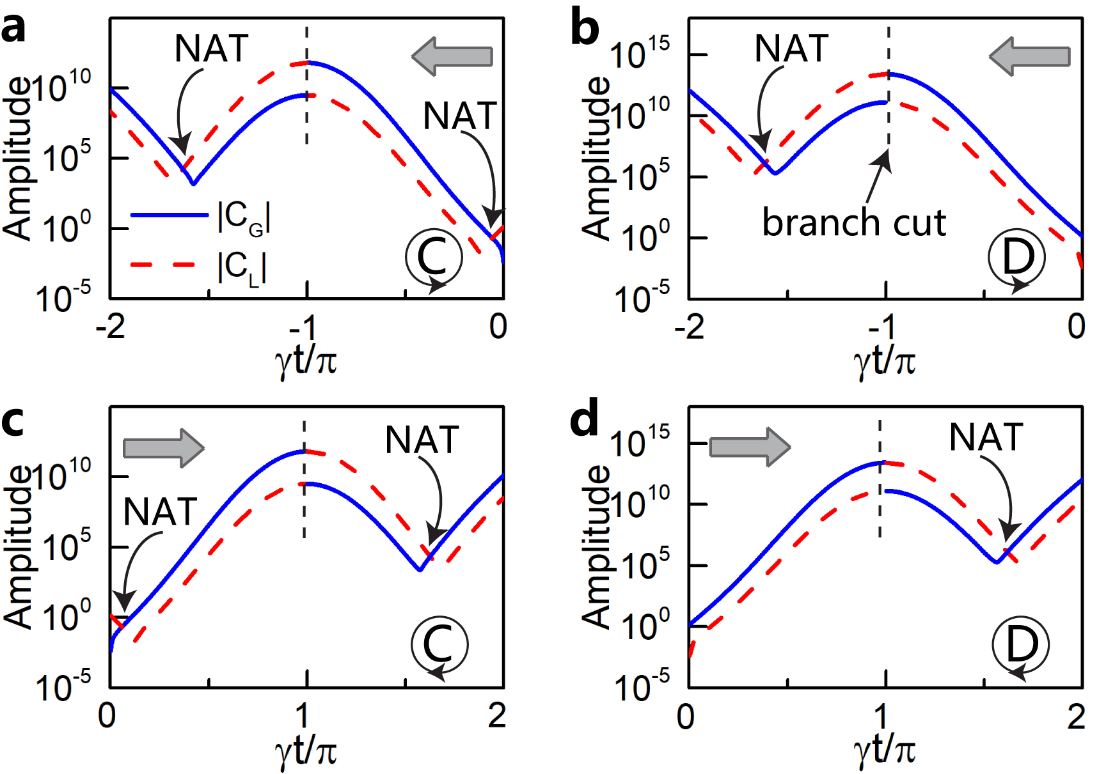


**Supplementary Figure 4. Non-chiral dynamics with the starting point in the *PT*-symmetric phase.** **a-b** Calculated amplitude coefficients for the anti-clockwise loops with **a** state C and **b** state D as the initial state. **c-d** Same as **a-b** except for clockwise loops.

**Supplementary References**

[S1] Hassan, A. U., Zhen, B., Soljačić, M., Khajavikhan, M. & Christodoulides, D. N. Dynamically encircling exceptional points: exact evolution and polarization state conversion. *Phys. Rev. Lett.* **118**, 093002 (2017).

[S2] Slater, L. J. *Confluent Hypergeometric Functions* (Cambridge University Press, Cambridge, England, 1960).

[S3] Uzdin, R., Mailybaev, A. & Moiseyev, N. On the observability and asymmetry of adiabatic state flips generated by exceptional points. *J. Phys. A Math. Theor.* **44**, 435302 (2011).

[S4] Berry, M. V. & Uzdin, R. Slow non-Hermitian cycling: exact solutions and the Stokes phenomenon. *J. Phys. A Math. Theor.* **44**, 435303 (2011).

[S5] Gilary, I., Mailybaev, A. A. & Moiseyev, N. Time-asymmetric quantum-state-exchange mechanism. *Phys. Rev. A* **88**, 010102 (2013).

[S6] Milburn, T. J. et al. General description of quasiadiabatic dynamical phenomena near exceptional points. *Phys. Rev. A* **92**, 052124 (2015).

[S7] Zhang, X. L., Wang, S. B., Hou, B. & Chan, C. T. Dynamically encircling exceptional points: in situ control of encircling loops and the role of the starting point. *Phys. Rev. X* **8**, 021066 (2018).
